# Investigating the causal effect of various metabolites on postherpetic neuralgia: a Mendelian randomization study

**DOI:** 10.3389/fneur.2024.1421670

**Published:** 2024-11-22

**Authors:** Jianyu Zhu, Jiahao Chen, Yuefen Zuo, Kun Song, Huilian Liao, Xianping Wu

**Affiliations:** ^1^Department of Clinical Medical Laboratory, Shunde Hospital of Guangzhou University of Chinese Medicine, Guangzhou, China; ^2^Department of Anesthesiology, Shunde Hospital of Guangzhou University of Chinese Medicine, Guangzhou, China; ^3^Department of Nursing, Shunde Hospital of Guangzhou University of Chinese Medicine, Guangzhou, China

**Keywords:** postherpetic neuralgia, Mendelian randomization, metabolite, toxicology, NAAG

## Abstract

**Background:**

Common side effect of Herpes Zoster, postherpetic neuralgia (PHN), causes persistent pain that seriously affects quality of life. Lack of dependable biomarkers makes the clinical diagnosis and treatment of PHN difficult, so complicating the assessment of therapeutic efficacy. Blood metabolites are becoming more and more well known as significant disease markers. With an aim to find possible biomarkers for diagnosis and treatment, this work investigates the causal link between blood metabolites and PHN using Mendelian randomization.

**Methods:**

This work evaluated causal relationships between PHN and 1,091 plasma metabolites using Mendelian randomization (MR). Complementing MR-Egger and weighted median approaches, the main causality analysis was done using inverse variance weighted (IVW) and Wald ratio (WR) approaches. Robustness was checked using sensitivity analyses including CAUSE, Cochran’s *Q* tests, leave-one-out analysis, MR-PRESSO, and MR-Egger intercept analysis. Reverse MR analysis and linkage disequilibrium score regression (LDSC) was used to assess significant correlations as well. Two-step MR analysis was also used to look at the mediating function of positively correlated metabolites in the causal pathway.

**Results:**

The results of this study indicated a significant association between *N*-acetyl-aspartyl-glutamate (NAAG) and PHN, with an odds ratio (OR) of 0.83 (95% CI: 0.76–0.91, *p* = 2.68E-05). Moreover, five potential associated metabolites were identified: Gamma-glutamylthreonine (OR = 1.60, 95% CI: 1.16–2.20, *p* = 0.004), 3-hydroxyphenylacetoylglutamine (OR = 1.43, 95% CI: 1.00–2.05, *p* = 0.048), Caprate (10:0) (OR = 1.86, 95% CI: 1.11–3.12, *p* = 0.018), X-12013 (OR = 1.64, 95% CI: 1.03–2.60, *p* = 0.035), and X-17328 (OR = 1.50, 95% CI: 1.04–2.18, *p* = 0.032). Additionally, NAAG likely acts as a complete mediator between FOLH1(CGPII) and postherpetic neuralgia in the causal pathway.

**Conclusion:**

The results of this study indicated a significant association between *N*-acetyl-aspartyl-glutamate (NAAG) and PHN, with an odds ratio (OR) of 0.83 (95% CI: 0.76–0.91, *p* = 2.68E-05). Furthermore five possible related metabolites were found: Glutamylthreonine gamma-wise (OR = 1.60, 95% CI: 1.16–2.20, *p* = 0.004), 3-hydroxyphenylacetoylglutamine (OR = 1.43, 95% CI: 1.00–2.05, *p* = 0.048), Caprate (10:0) (OR = 1.86, 95% CI: 1.11–3.12, *p* = 0.018), X-12013 (OR = 1.64, 95% CI: 1.03–2.60, *p* = 0.035), and X-17328 (OR = 1.50, 95% CI: 1.04–2.18, *p* = 0.032). Furthermore, in the causal pathway NAAG most certainly serves as a complete mediator between FOLH1(CGPII) and postherpetic neuralgia.

## Introduction

The viral disease herpes zoster (HZ) results from reactivation of latent varicella-zoster virus (VZV) within cranial or dorsal root nerves (1). Affecting almost 20% of persons diagnosed with herpes zoster, postherpetic neuralgia (PHN) is the most frequent and severe complication of HZ (2, 3). Any population can have PHN, although some risk factors greatly raise its frequency. With older people, especially at risk, age and immunosuppression are acknowledged as main causes of PHN frequency. The likelihood of PHN increases with age as well. People with severe immunosuppressive diseases are said to be more susceptible to PHN (4, 5). Immunosuppression reduces the ability of the immune system to control VZV immunosuppression can lead to a weakened immune response, which is critical in controlling the reactivation, so increasing of the incidence VZV, the causative agent of herpes zoster (shingles) and hence subsequently PHN (6). VZV reactivates when the immune system is compromised, VZV can reactivate more easily in immunocompromised people, increasing their risk of HZ and all-around effects, leading to a higher incidence of herpes zoster and its complications, including PHN (7, 8). Chronic, severe, and recurrent neuropathic pain defines PHN and can greatly compromise patients’ quality of life. Studies have found that PHN is linked to a higher risk of anxiety, depression, even suicide (9). PHN has a major effect, yet present, diagnostic tools are still inadequate. Usually based on the patient’s medical history and clinical signs of shingles, diagnosis depends on no clear biological markers or auxiliary tests especially for PHN (10). Examining exploring the relationship between blood metabolites and PHN could give may provide important new perspectives on insights for clinical diagnosis and treatment. Recent two studies predicted PHN risk using machine learning models to predict PHN risk have combined particular incorporated specific blood metabolite levels as predictive elements into their evaluation conditions (11, 12). Transcriptional another study conducting transcriptional analysis of on blood samples from PHN patients revealed found that changes in lipid, polysaccharide, and nucleotide metabolism as linked to are associated with PHN development (13). These results underline studies highlight the great possibilities vast potential of blood metabolites in forward PHN research. Currently, the treatment of PHN primarily involves the use of tricyclic antidepressants, pregabalin, gabapentin, and lidocaine patches as first-line therapies. If these treatments are ineffective, second- and third-line therapies, such as tramadol, capsaicin creams, and patches, may be considered (14). However, these treatments do not provide relief for all patients with PHN. Although the varicella-zoster virus (VZV) vaccine has demonstrated efficacy in preventing herpes zoster and thereby reducing the risk of PHN, and vaccines such as VZV live attenuated vaccine have begun to be administered in some countries and regions, the vaccination rate (9) remains suboptimal in some regions, and the cost-effectiveness of the VZV vaccine has been debated. PHN is still causing suffering to countless patients and causing a subject of debate. As PHN continues to afflict numerous patients and impose a substantial economic burden on healthcare systems, improving the level of clinical management and treatment of PHN remains an urgent priority.

In recent years, Mendelian randomization (MR) has become a potent tool for investigating disease etiology, gaining prominence in recent years providing a strong means to handle challenging biological and epidemiological problems (15). Without randomized controlled trials, MR offers compelling proof of causal links between exposures and outcomes (16). MR studies replace conventional exposure factors with genetic proxies using single nucleotide polymorphisms (SNPs) as instrumental variables to probe causal relationships with disease outcomes. Meiosis’s random distribution of alleles guarantees that genotypes are established before disease starts, so reducing the issues of reverse causality and confusing outcomes seen in observational studies (17).

Extensive genotyping of biological elements including circulating metabolites at the protein level has made possible by developments in high-throughput sequencing technologies (18). This has helped many SNPs to be found as well as an increasing corpus of research proving the relationships between blood metabolites and different diseases (19).

Aiming to fully evaluate the causal effects of 1,091 blood metabolites on PHN, in this work we use genome-wide association study (GWAS) summary data to perform MR analysis. This work aims to progress clinical diagnostics and therapeutic approaches for PHN by exploring the genetic and protein-level biological processes underlie of PHN.

## Materials and methods

### Study design

**Figure 1:**
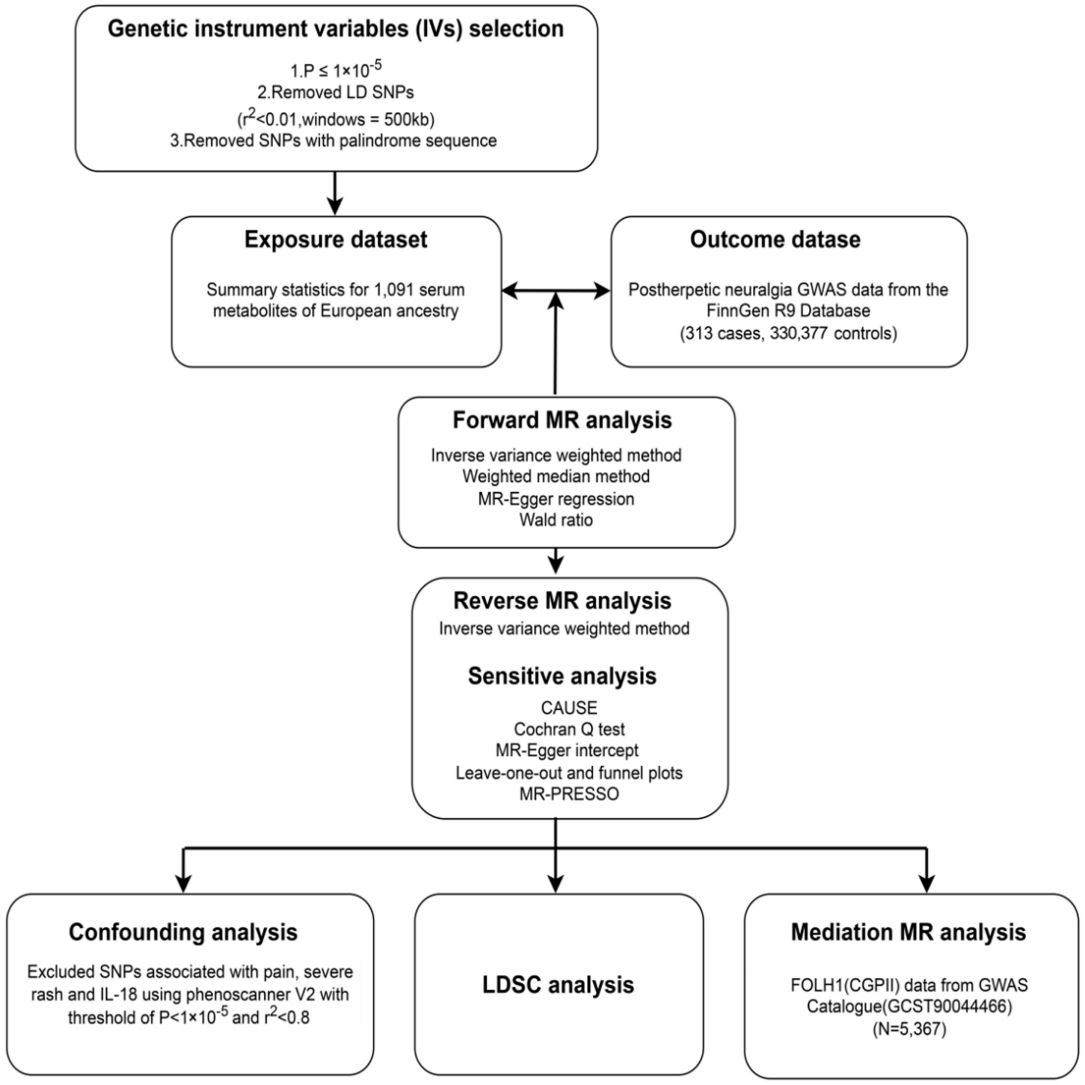
Overview of this Mendelian randomization (MR) study.

The general study design is shown in Figure 1, so offering a whole picture of the research framework. The next sections contain thorough explanations of the applied techniques and the traits of the study subjects. Three basic presumptions underlie the Mendelian randomization (MR) analysis in this study: (a) the instrumental variables (IVs) must be significantly associated with the exposures (20); (b) the IVs should not be associated with any confusing factors (20); (c) the IVs must influence the outcomes just by means of the exposures, without any direct or other indirect pathway (20).

All statistical calculations were carried out using the R software (version 4.2.3) was used in all statistical analyses. Two Sample MR (version 0.5.7) and CAUSE (version 1.2.0) packages were especially used for the MR and sensitivity studies.

### The source of GWAS summary data

Chen’s work provides the summary data on circulating metabolites used in this study, comprising a total of 1,091 specific metabolites subjected to strict quality control measures (21). Out of these metabolites, 195 are still unknown while 896 are known. All from people with European heritage, these metabolites underwent genome-wide association analysis (Supplementary Table S1). Based on their chemical characteristics and biological roles, 850 metabolites were arranged analytically into eight main classes. Among these are lipids, amino acids, xenobiotics, nucleotides (Table 1), cofactors and vitamin supplements, carbohydrates, peptides, and energy (21). The remaining 241 metabolites left were classified as either unknown or only somewhat defined molecules (21).

Source of the GWAS summary data for postherpetic neuralgia was the R9 release of the FinnGen consortium (22). With a dataset comprising 313 PHN cases and 330,377 controls, PHN cases were found using the International Classification of Diseases, 10th Revision (ICD-10) diagnostic code (G53.0, postzoster neuralgia).

Furthermore derived from the GWAS Catalogue (GCST90089) were summary data for serum levels of the protein FOLH1 (CGPII). From Gudjonsson et al. (23), this proteogenomic dataset comprises 2,091 serum proteins investigated in 5,367 European-ancestral individuals with European ancestry from Gudjonsson’s study (Table 1).

**Table 1:** Outlined the attributes of all summary genome-wide association studies performed in this Mendelian randomization investigation.

Trait	Sample size	Ancestry	Dataset source	Year
Blood metabolites	4,098 ~ 8,299	European	GWAS Catalog	2023
Postherpetic neuralgia	330,690	European	FinnGen R9	2023
FOLH1 (CGPII)	5,367	European	GWAS Catalog	2022

### Selection of instrumental variables

We used rigorous criteria for selecting instrumental variables (IVs), requiring a significance level of *p* < 1 × 10^−5^ for identified causally associated metabolites and *p* < 5 × 10^−8^ for mediation MR analysis, to choose genetic instruments for MR analysis. Figure 1 shows the choosing process, which consists and comprises the following phasteps: (1) Linkage disequilibrium (LD) analysis using the European (EUR) 1,000 Genomes Project Phase 3 reference panel guaranteed the choice of independent IVs with a physical distance within 500 kb and an LD parameter (*r*^2^) of less than 0.01. (2) We kept only SNPs with alleles matching those of the exposure and outcome data were kept. (3) Strict criteria were developed to guarantee that just strong IVs would be included, requiring an F-statistic of a minimum of 10. (4) The IVs selected for the exposure variable were verified to have no direct relationship to the outcome, hence a *p*-value higher than 5 × 10^−8^ was needed.

### MR analysis for metabolites causally associated to postherpetic neuralgia

Potential relationships between 1,091 metabolites and PHN were investigated using a two-sample MR study. The study followed exactly the guidelines of STROBE-MR for reporting rules and completed the relevant checklist was completed for this study (Supplementary Table S5) (24).

While the inverse variance weighting (IVW) method was used in cases involving several instrumental variables (IV), the Wald ratio (WR) method was applied for the forward MR analysis when only a single IV was available. The main method for estimating causal effects in MR studies is the IVW method, which combines ratio estimates from every SNP to find the total causal effect as GWAS summary data usually comprises many uncorrelated variants.

Reverse MR analysis was also done to see if reverse causation affected the noted causal relationships. The reverse (25) MR analysis followed the same IV selection criteria and clumping preferences from the forward MR analysis to conduct reverse MR analysis.

For the forward MR analysis, we set a conservative Bonferroni threshold for statistical significance at *p* < 0.05/1,091, where 1,091 represents the total number of metabolites finally included into the MR analysis, so minimizing the risk of false positive results in multiple testing (26). When uncorrected *P*_IVW_ was <0.05 and beta values of two other methods [MR-Egger (27), weighted median (28)] were in the same direction of IVW method (29), suggestive associations were taken under consideration. The metabolite was significantly linked with postherpetic neuralgia when the *P*_IVW_ following Bonferroni correction was <0.000045 (0.05 divided by 1,091).

### Sensitivity analysis

Using five analytical approaches, CAUSE (30), MR-PRESSO (31, 32), MR-Egger intercept (27), Cochran’s *Q* test (33), and leave-one-out analysis (LOO) (34) suggestive and significant results in the Mendelian randomization (MR) analysis were sensitivity analyzed. Leveraging independent SNPs to improve detection power helps the CAUSE is an MR method, based on Bayesian posterior probabilities, to lower the risk of false positives arising from correlated and uncorrelated horizontal pleiotropy (30). A new tool in MR analysis, MR-PRESSO method, a novel approach in MR analysis, was used to identify and fix horizontal pleiotropic outliers, so guaranteeing more accurate effect estimations. Further assessing the existence of horizontal pleiotropy over the dataset, the MR-PRESSO global test (31, 32). The MR-PRESSO global test was applied to assess the presence of horizontal pleiotropy. The MR-Egger intercept was computed to find and explain horizontal pleiotropy, so reducing possible biases brought about by invalid instrumental variables (27). Variance among SNPs was evaluated using Cochran’s *Q* test, so revealing the consistency of the genetic tools (33). Finally, a leave-one-out (LOO) study was conducted to investigate the effect of individual SNPs on the general results, so guaranteeing the validity of the conclusions (34).

### Confounding analysis

The sensitivity study included an evaluation of several statistical techniques to check possible deviations from the MR assumptions. We also used the Phenoscanner V2 (Supplementary Table S4) to investigate whether the SNPs linked with positive metabolites also displayed correlations with common risk factors that could possibly bias the MR estimates (35), such as pain in the herpes stage (36), severe rash (36) and IL-18 (37). Should any confounders be found, the MR study will be repeated following the exclusion of these SNPs to validate the findings.

### Evaluation of genetic correlation

In cases when there is a genetic correlation between traits, several studies have shown that Mendelian randomization (MR) frequently produces false positive results in cases where there is a genetic correlation between traits (38, 39). Although SNPs linked to postherpetic neuralgia were ruled out during instrument selection of instruments, there is still a chance that a mix of SNPs, which individually show no significant association, may still affect the genetic inclination to postherpetic neuralgia. We thus assess the genetic correlation between found metabolites and postherpetic neuralgia using LDSC in order to look at whether shared genetic architecture influences the established causal relationship is influenced by shared genetic architecture.

### Mediation MR analysis

We searched for proteins connected to significantly related metabolites (*P*_bonferroni_ < 0.05) using NBCI and PubMed databases to have a complete knowledge of their exact function in the development and progression of postherpetic neuralgia (PHN). After that, a two-step Mendelian randomization (MR) study was carried out to investigate whether the found metabolites could potentially mediate the link between particular proteins and PHN (40).

The initial step entailed quantifying the causal effect (*β*_XZ_) of the genetically identified protein on positive metabolites through UVMR. The second step evaluated the causal impact (*β*_ZY_) of these beneficial metabolites on postherpetic neuralgia. When evidence suggested that the identified protein affected positive metabolites, which subsequently influenced positive metabolites, which in turn impacted the outcome, the “product of coefficients” method was utilized to assess the mediating effect (*β*_XY’_ = *β*_XZ_ × *β*_ZY_) of the identified protein on postherpetic neuralgia via positive metabolites. The standard errors for these mediation effects were calculated utilizing the delta method.

## Results

### Selection of IVs for MR analysis

The Mendelian randomization (MR) estimation for postherpetic neuralgia included 1,091 metabolites overall. For 1,091 metabolites, the count of instrumental variables (IVs) ranged from 11 to 102. With a minimum value of 19.506, all of these IVs displayed F statistics higher than 10, so indicating the sufficient effectiveness of the IVs for all 1,091 metabolites in the MR analysis (see Supplementary Table S2).

### Forward MR analysis for associated metabolites for postherpetic neuralgia

Only results with a PIVW were 0.05 and beta values of two other methods (MR-Egger, weighted median) are in the same direction of IVW method were included in the final study results, so guaranteeing the robustness of the results.

**Figure 2:**
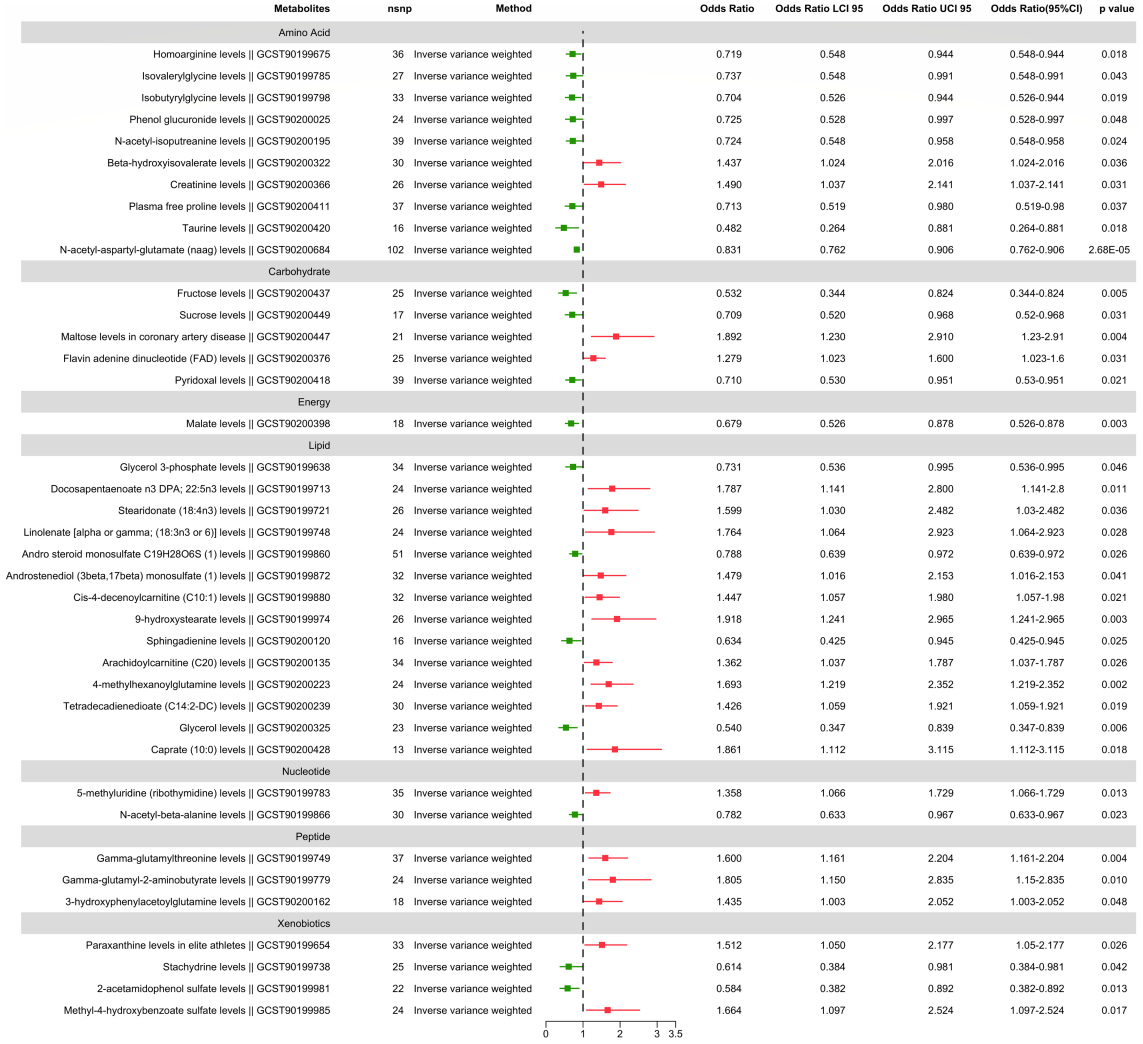
The main results of forward MR analysis.

**Figure 3:**
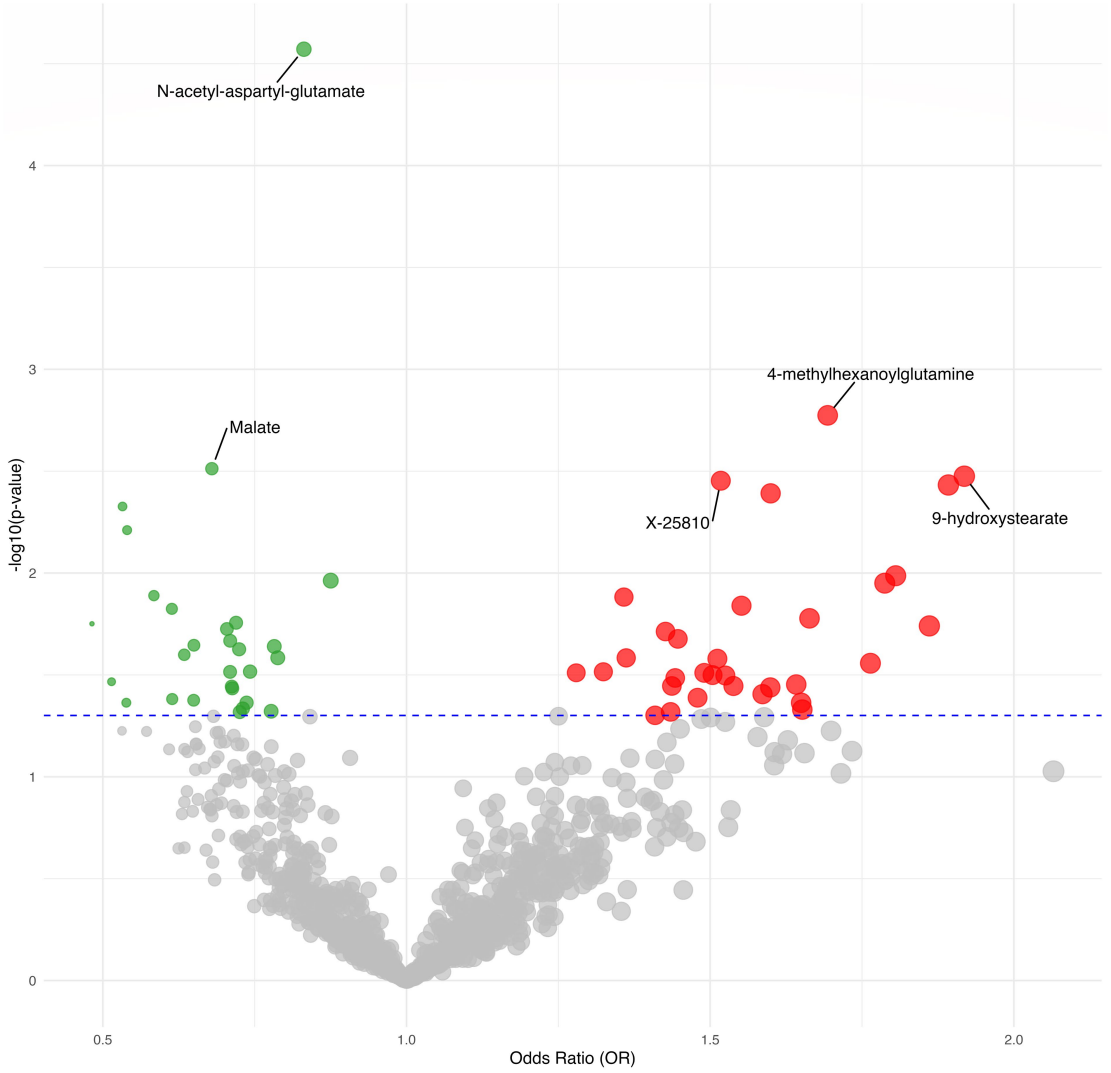
Volcano plot depicting the forward MR results.

We found suggestive correlations for postherpetic neuralgia in 56 metabolites (38 known metabolites and 18 unknown metabolites), as shown in Figures 2, 3, while one known metabolite showed a significant association. Among the 56 suggestive associations, Glycerol 3-phosphate (OR = 0.73, 95%CI = 0.54–0.99, *p* = 0.046), Homoarginine (OR = 0.72, 95%CI = 0.55–0.94, *p* = 0.018), Stachydrine (OR = 0.61, 95%CI = 0.38–0.98, *p* = 0.042), Isovalerylglycine (OR = 0.74, 95%CI = 0.55–0.99, *p* = 0.043), Isobutyrylglycine (OR = 0.70, 95%CI = 0.53–0.94, *p* = 0.019), Andro steroid monosulfate C19H28O6S (1) (OR = 0.79, 95%CI = 0.64–0.97, *p* = 0.026), *N*-acetyl-beta-alanine (OR = 0.78, 95%CI = 0.63–0.97, *p* = 0.023), 2-acetamidophenol sulfate (OR = 0.58, 95%CI = 0.38–0.89, *p* = 0.013), Phenol glucuronide (OR = 0.73, 95%CI = 0.53–1.00, *p* = 0.048), Sphingadienine (OR = 0.63, 95%CI = 0.43–0.94, *p* = 0.025), *N*-acetyl-isoputreanine (OR = 0.72, 95%CI = 0.55–0.96, *p* = 0.024), Glycerol (OR = 0.54, 95%CI = 0.35–0.84, *p* = 0.006), Malate (OR = 0.68, 95%CI = 0.53–0.88, *p* = 0.003), Plasma free proline (OR = 0.71, 95%CI = 0.52–0.98, *p* = 0.037), Pyridoxal (OR = 0.71, 95%CI = 0.53–0.95, *p* = 0.022), Taurine (OR = 0.48, 95%CI = 0.26–0.88, *p* = 0.018), Fructose (OR = 0.53, 95%CI = 0.34–0.82, *p* = 0.005), Sucrose (OR = 0.71, 95%CI = 0.52–0.97, *p* = 0.031), X-12127 (OR = 0.71, 95%CI = 0.52–0.98, *p* = 0.036), X-12729 (OR = 0.88, 95%CI = 0.79–0.97, *p* = 0.011), X-21319 (OR = 0.54, 95%CI = 0.30–0.98, *p* = 0.043), X-19438 (OR = 0.61, 95%CI = 0.41–0.91, *p* = 0.015), X-21285 (OR = 0.74, 95%CI = 0.57–0.97, *p* = 0.030), X-22520 (OR = 0.65, 95%CI = 0.45–0.94, *p* = 0.023) and X-25810 (OR = 1.52, 95%CI = 1.15–2.01, *p* = 0.0035) were suggestive associated with a reduced risk of postherpetic neuralgia.

Conversely, Paraxanthine in elite athletes (OR = 1.51, 95%CI = 1.05–2.18, *p* = 0.026), Docosapentaenoate n3 DPA; 22:5n3 (OR = 1.79, 95%CI = 1.14–2.80, *p* = 0.011), Stearidonate (18:4n3) (OR = 1.60, 95%CI = 1.03–2.48, *p* = 0.036), Linolenate [alpha or gamma; (18:3n3 or 6)] (OR = 1.76, 95%CI = 1.06–2.92, *p* = 0.028), Gamma-glutamylthreonine (OR = 1.60, 95%CI = 1.16–2.20, *p* = 0.004), Gamma-glutamyl-2-aminobutyrate (OR = 1.81, 95%CI = 1.15–2.84, *p* = 0.010), 5-methyluridine (ribothymidine) (OR = 1.36, 95%CI = 1.07–1.73, *p* = 0.013), Androstenediol (3beta, 17beta) monosulfate (1) (OR = 1.48, 95%CI = 1.02–2.15, *p* = 0.041), Cis-4-decenoylcarnitine (C10:1) (OR = 1.45, 95%CI = 1.06–1.98, *p* = 0.021), 9-hydroxystearate (OR = 1.92, 95%CI = 1.24–2.96, *p* = 0.003), Methyl-4-hydroxybenzoate sulfate (OR = 1.66, 95%CI = 1.10–2.52, *p* = 0.017), Arachidoylcarnitine (C20) (OR = 1.36, 95%CI = 1.04–1.79, *p* = 0.026), 3-hydroxyphenylacetoylglutamine (OR = 1.43, 95%CI = 1.00–2.05, *p* = 0.048), 4-methylhexanoylglutamine (OR = 1.69, 95%CI = 1.22–2.35, *p* = 0.002), Tetradecadienedioate (C14:2-DC) (OR = 1.43, 95%CI = 1.06–1.92, *p* = 0.019), Beta-hydroxyisovalerate (OR = 1.44, 95%CI = 1.02–2.02, *p* = 0.036), Creatinine (OR = 1.49, 95%CI = 1.04–2.14, *p* = 0.031), Flavin adenine dinucleotide (FAD) (OR = 1.28, 95%CI = 1.02–1.60, *p* = 0.031), Caprate (10:0) (OR = 1.86, 95%CI = 1.11–3.12, *p* = 0.018), Maltose in coronary artery disease (OR = 1.89, 95%CI = 1.23–2.91, *p* = 0.004), X-11849 (OR = 1.54, 95%CI = 1.03–2.30, *p* = 0.036), X-11850 (OR = 1.65, 95%CI = 1.02–2.68, *p* = 0.043), X-12117 (OR = 1.32, 95%CI = 1.03–1.71, *p* = 0.031), X-12013 (OR = 1.64, 95%CI = 1.03–2.60, *p* = 0.035), X-12818 (OR = 1.44, 95%CI = 1.03–2.02, *p* = 0.033), X-13684 (OR = 1.41, 95%CI = 1.00–1.99, *p* = 0.050), X-17328 (OR = 1.50, 95%CI = 1.04–2.18, *p* = 0.032), X-23639 (OR = 1.55, 95%CI = 1.09–2.21, *p* = 0.014), X-23587 (OR = 1.59, 95%CI = 1.02–2.46, *p* = 0.039) and X-25810 (OR = 1.52, 95%CI = 1.15–2.01, *p* = 0.004) were suggestive associated with an increased risk of postherpetic neuralgia.

Furthermore found to be significantly linked with a lower risk of postherpetic neuralgia was *N*-acetyl-aspartyl-glutamate (NAAG), (OR = 0.83, 95%CI = 0.76–0.91, *p* = 2.68E-05) which passed multiple corrections and was found to be significantly associated with a reduced risk of postherpetic neuralgia.

### Reverse MR analysis for associated metabolites for postherpetic neuralgia

Except from X-19438 (*P*_IVW_ = 0.029), Arachidoylcarnitine (C20) (*P*_IVW_ = 0.031), and N-acetyl-isoputreanine (*P*_IVW_ = 0.006), the results of reverse Mendelian randomization (MR) analysis show no evidence of a reverse causal relationship between the metabolites significantly and suggestively linked with postherpetic neuralgia identified in the forward MR analysis. Further information is supplied in Supplementary Table S3.

### Sensitivity analysis

Only six metabolites (Gamma-glutamylthreonine *P_CAUSE_* = 0.048, 3-hydroxyphenylacetoylglutamine *P_CAUSE_* = 1.18E-07, Caprate 10:0 *P_CAUSE_* = 0.006, X-12013 *P_CAUSE_* E = 0.012, X-17328 *P_CAUSE_* = 0.011, *N*-acetyl-aspartyl-glutamate [NAAG] *P_CAUSE_* = 0.010) kept a significant association in the causal model, according to the CAUSE analysis results. No correlated or uncorrelated pleiotropic effects were observed. In the causal model of CAUSE (PCA USE > 0.05, Table 2), the remaining 57 metabolites might have either correlated or uncorrelated pleiotropic effects in the causal model of CAUSE (*P_CAUSE_* > 0.05, see Table 2).

**Table 2:** MR sensitive estimates for the association between identified metabolites and postherpetic neuralgia.

Exposure	Heterogeneity (Cochran’s *Q* test)		MR-Egger intercept			MR-PRESSO	CAUSE	
	Q_IVW_	*P_Q_IVW_*	Intercept	SE	*P*	*P_global test_*	SE	*P_CAUSE_*
Glycerol 3-phosphate	31.2414	0.5548	0.0460	0.0468	0.3336	0.5140	0.2178	1.0000
Paraxanthine in elite athletes	26.5279	0.7398	−0.0506	0.0483	0.3026	0.7600	0.8278	0.6723
Homoarginine	29.5083	0.7302	−0.0127	0.0395	0.7505	0.6560	0.1866	0.6400
Docosapentaenoate n3 DPA; 22:5n3	24.7559	0.3630	−0.0532	0.0601	0.3850	0.3940	0.5030	0.8163
Stearidonate (18:4n3)	16.0649	0.9129	0.0521	0.0507	0.3146	0.9210	0.4918	0.2462
Stachydrine	21.7851	0.5921	0.0711	0.0557	0.2145	0.6040	2.5254	0.5113
Linolenate [alpha or gamma; (18:3n3 or 6)]	16.0716	0.8522	−0.0224	0.0694	0.7498	0.8630	0.8520	1.0000
Gamma-glutamylthreonine	27.6892	0.8381	0.0150	0.0381	0.6968	0.8640	0.3428	0.0479
Gamma-glutamyl-2-aminobutyrate	29.0962	0.1771	0.0163	0.0627	0.7971	0.2080	1.7534	0.2817
5-methyluridine (ribothymidine)	37.2691	0.3211	0.0129	0.0353	0.7175	0.4070	0.1294	0.2786
Isovalerylglycine	27.1313	0.4025	0.0894	0.0434	0.0498	0.4620	0.3572	0.9900
Isobutyrylglycine	26.3452	0.7481	0.0141	0.0423	0.7412	0.7810	0.2339	0.3878
Andro steroid monosulfate C19H28O6S (1)	38.8542	0.8733	0.0096	0.0281	0.7336	0.8920	0.1141	0.6612
*N*-acetyl-beta-alanine	22.0829	0.8167	0.0357	0.0321	0.2757	0.8330	0.2052	0.0790
Androstenediol (3beta, 17beta) monosulfate (1)	28.6803	0.5859	−0.0522	0.0372	0.1705	0.6020	0.2952	0.4392
*Cis*-4-decenoylcarnitine (C10:1)	41.9005	0.0915	0.0034	0.0568	0.9523	0.1090	0.1728	0.1905
9-hydroxystearate	20.6285	0.7131	0.0619	0.0705	0.3888	0.7130	4.5512	0.1425
2-acetamidophenol sulfate	12.4375	0.9270	−0.0039	0.0574	0.9464	0.9380	3.1092	0.7751
Methyl-4-hydroxybenzoate sulfate	16.4650	0.8347	−0.0589	0.0572	0.3149	0.8320	6.6203	0.1804
Phenol glucuronide	23.9397	0.4072	0.0170	0.0525	0.7485	0.3610	1.3051	0.9994
Sphingadienine	15.3926	0.4235	0.0544	0.0768	0.4905	0.4230	8.2829	0.3728
Arachidoylcarnitine (C20)	31.2739	0.5532	−0.0323	0.0361	0.3771	0.5760	0.2158	0.9583
3-hydroxyphenylacetoylglutamine	15.8489	0.5346	−0.0194	0.0613	0.7553	0.6060	3.8835	1.18E-07
*N*-acetyl-isoputreanine	35.1169	0.6035	0.0048	0.0387	0.9017	0.6340	0.1286	0.8430
4-methylhexanoylglutamine	26.0208	0.2999	−0.0385	0.0503	0.4518	0.3100	0.2878	0.1901
Tetradecadienedioate (C14:2-DC)	28.4762	0.4926	−0.0446	0.0432	0.3116	0.5340	0.2188	0.4538
Beta-hydroxyisovalerate	15.4102	0.9815	0.0237	0.0500	0.6388	0.9920	0.2193	0.4364
Glycerol	26.8911	0.2154	−0.0302	0.0469	0.5260	0.2710	2.0633	1.0000
Creatinine	18.8598	0.8038	−0.0802	0.0425	0.0712	0.6810	1.7709	0.8278
Flavin adenine dinucleotide (FAD)	15.2744	0.9125	0.0052	0.0406	0.8984	0.8790	0.1553	0.0696
Malate	10.8160	0.8660	0.0197	0.0374	0.6068	0.9000	0.3072	0.8541
Plasma free proline	42.1405	0.2225	−0.0095	0.0492	0.8480	0.2500	0.2456	0.6207
Pyridoxal	28.6621	0.8634	0.0898	0.0433	0.0448	0.8690	0.5758	0.9999
Taurine	18.4622	0.2391	0.0635	0.0780	0.4288	0.2630	5.7526	0.8020
Caprate (10:0)	7.2587	0.8401	0.0147	0.0645	0.8237	0.8910	5.9329	0.0065
Fructose	24.5656	0.4297	−0.0833	0.0618	0.1910	0.4210	1.4387	0.8190
Maltose in coronary artery disease	11.1360	0.9426	−0.0434	0.0583	0.4652	0.9390	4.4110	0.2326
Sucrose	16.3404	0.4295	−0.0119	0.0424	0.7828	0.5550	4.4520	0.0529
X-11849	38.3945	0.1399	−0.0325	0.0657	0.6241	0.1530	0.4440	0.4743
X-11850	28.4711	0.1606	−0.1216	0.0663	0.0809	0.1940	3.5370	0.8117
X-12127	35.4638	0.5411	0.0035	0.0585	0.9526	0.5460	4.8787	1.0000
X-12117	35.3390	0.4998	−0.0269	0.0351	0.4490	0.4470	0.1492	0.7208
X-12013	31.8502	0.0606	0.0292	0.0650	0.6586	0.0740	3.0723	0.0116
X-12729	29.3953	0.2934	0.0389	0.0340	0.2639	0.4100	3.1904	0.3564
X-12818	15.9807	0.7178	−0.0628	0.0493	0.2181	0.7420	5.2094	0.9983
X-13684	40.9683	0.2251	0.0811	0.0452	0.0813	0.2350	0.2015	0.4800
X-17328	21.8620	0.5286	0.0415	0.0658	0.5351	0.5480	3.8020	0.0108
X-21319	8.8792	0.7132	0.0311	0.0825	0.7136	0.7430	0.4856	0.9514
X-19438	14.5679	0.9095	0.0448	0.0505	0.3844	0.9190	0.7856	0.2819
X-21285	30.3622	0.7333	−0.0080	0.0424	0.8520	0.7060	0.2108	0.2904
X-23639	17.5983	0.6139	0.0550	0.0456	0.2428	0.5830	0.3062	0.4802
X-22520	12.0783	0.8822	−0.0331	0.0708	0.6455	0.8840	0.2638	0.6571
X-23587	23.7995	0.4150	0.0307	0.0654	0.6438	0.4050	5.5074	0.9832
X-24951	9.5943	0.9749	0.0280	0.0579	0.6338	0.9720	0.7479	0.9989
X-25790	7.7685	0.9010	0.1139	0.1056	0.3002	0.9140	0.3822	0.9997
X-25810	22.1363	0.9553	−0.0241	0.0360	0.5079	0.9630	0.4853	0.8116
*N*-acetyl-aspartyl-glutamate (naag)	97.9126	0.5685	−0.0087	0.0289	0.7631	0.6210	0.0448	0.0104

The relevant SNPs for positive metabolites showed no heterogeneity according to Cochran’s *Q* test (all *P*_Q_IVW_ > 0.05, see Table 2).

For two metabolites the MR-Egger intercept analysis revealed horizontal pleiotropy for two metabolites (Isovalerylglycine *p* = 0.0498; Pyridoxal *p* = 0.0448), for the other metabolites, no horizontal pleiotropy was seen (*p* > 0.05, see Table 2).

Regarding all positively correlated metabolites (*P_global test_* > 0.05, see Table 2), the MR-PRESSO analysis revealed no indication of horizontal pleiotropy.

**Figure 4:**
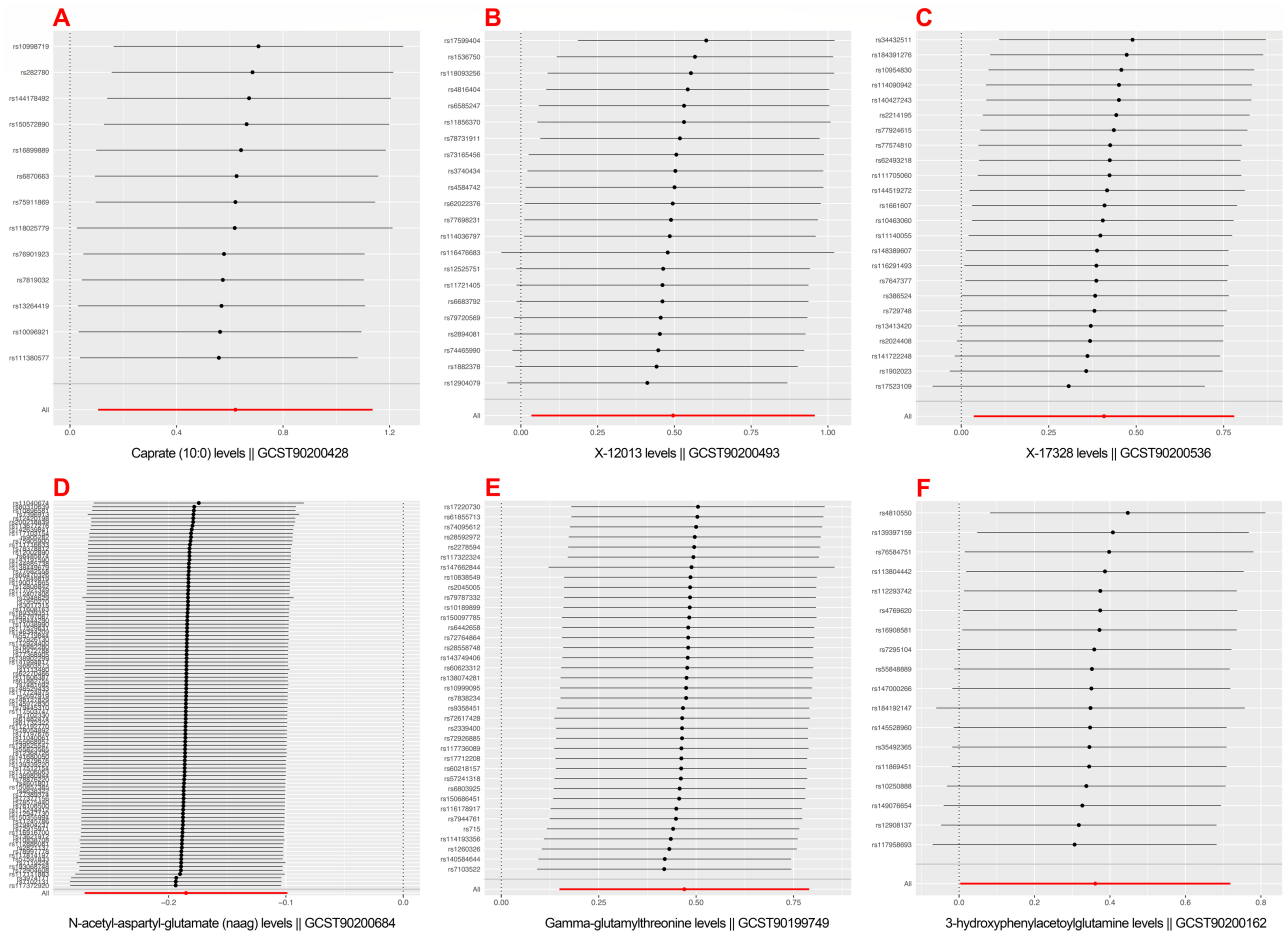
Mendelian randomization leave-one-out sensitivity analysis for positively associated metabolites. (A) Caprate (10:0); (B) X-12013; (C) X-17328; (D) *N*-acetyl-aspartyl-glutamate (NAAG); (E) Gamma-glutamylthreonine; (F) 3-hydroxyphenylacetoylglutamine.

Positively correlated metabolites did not differ significantly in their estimated causal effects on postherpetic neuralgia, according the leave-one-out (LOO) analysis (see Figure 4).

**Figure 5:**
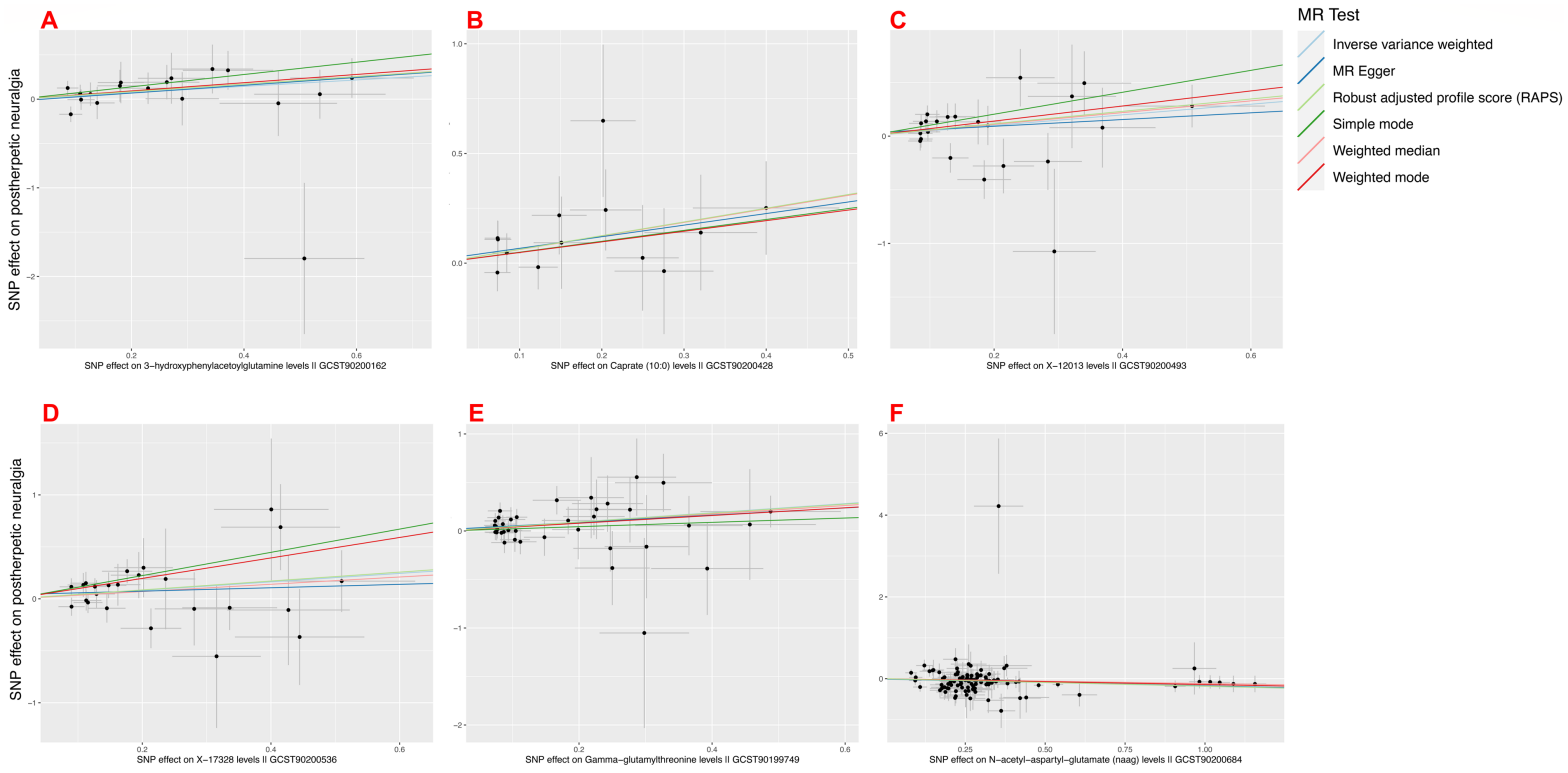
Mendelian randomization scatter plot of positively associated metabolites passing sensitivity analysis. (A) 3-hydroxyphenylacetoylglutamine; (B) Caprate (10:0); (C) X-12013; (D) X-17328; (E) Gamma-glutamylthreonine; (F) *N*-acetyl-aspartyl-glutamate (NAAG).

On postherpetic neuralgia, we thus solely included the MR analyses results of Gamma-glutamylthreonine, 3-hydroxyphenylacetoylglutamine, Caprate (10:0), X-12013, X-17328, *N*-acetyl-aspartyl-glutamate (NAAG) on postherpetic neuralgia (see Figure 5).

### Confounding analysis

A confounding analysis was performed on Gamma-glutamylthreonine, 3-hydroxyphenylacetylglutamine, Caprate (10:0), X-12013, X-17328, and *N*-acetyl-aspartyl-glutamate (NAAG) underwent a confusing analysis. Among these positive metabolites, no confining SNPs were found in Supplementary Table S4 for more detail.

### Evaluation of genetic correlation

The LDSC study turned up no metabolites with a genetic link to postherpetic neuralgia. Particularly, estimates derived from LDSC-based models showed little genetic correlation between postherpetic neuralgia and *N*-acetyl-aspartyl-glutamate (NAAG) (Rg = 0.137, Se = 0.279, *p* = 0.624). This suggests that the MR estimates are not influenced by shared genetic components have little effect on the MR estimations. These results verify the absence of confusing by shared genetic components and help to support the dependability of the MR estimates.

Due to restrictions in the sample size or heritability, the LDSC analysis could not be performed for five metabolites (Gamma-glutamylthreonine, 3-hydroxyphenyl acetylglutamine, Caprate (10:0), X-12013, X-17328) in postherpetic neuralgia due to limitations in the sample size or heritability.

### Mediation MR analysis

In forward MR analysis, *N*-acetyl-aspartyl-glutamate (NAAG) was the only metabolite clearly linked (*P*_bonferroni_ < 0.05) with postherpetic neuralgia. Additional NCBI and PubMed research turned up evidence showing FOLH1 acts as a glutamate carboxypeptidase on several substrates, including the nutrient folate and the neuropeptide NAAG and the nutrient folate. It is expressed in kidneys, central and peripheral nervous systems, and the prostate among other tissues.

The mechanism of the causal pathway mediated by NAAG in the actions of FOLH1 and PHN is yet unknown. Between FOLH1 and PHN, we also performed UVMR and discovered no causal relationship between them (*P*_IVW_ = 0.496, *β*_XY_ = 0.117). Therefore, a two-step MR study was conducted to investigate whether NAAG modulates the association between FOLH1 and postherpetic neuralgia.

UVMR analysis gave reliable proof in the first step that reduced risk of NAAG (*P*_IVW_ = 1.81E-07, *β*_XZ_ = −1.692) was much correlated with genetically predicted FOLH1. Although Cochran’s *Q* test found heterogeneity (*P*_Q_IVW_ < 0.05) MR-Egger intercept analysis revealed no horizontal pleiotropy (*P*_intercept_ > 0.05), although Cochran’s *Q* test indicated the presence of heterogeneity (*P*_Q_IVW_ < 0.05). To faithfully estimate the causal effects, a random-effects model was used, so preserving consistency in the MR results (Table 3).

**Table 3:** UVMR estimate mediation effect of NAAG between FOLH1(CGPII) and postherpetic neuralgia.

Pathway	Exposure	Outcome	Causal effect measurement (IVW)		Heterogeneity (Cochran’s *Q* test)		Pleiotropy (MR-Egger)	
			Beta (95%CI)	*P*_IVW_	*Q*_IVW_	*P*_Q_IVW_	Intercept	*P*_intercept_
XZ	FOLH1(CGPII)	NAAG	−1.692346 (−2.328076, −1.056617)	1.81E-07	411.882	7.53E-88	0.225	0.137
ZY	NAAG	PHN	−0.1879514 (−0.2773819, −0.09852097)	3.80E-05	51.474	0.888	−0.040	0.277
XY	FOLH1(CGPII)	PHN	0.1171362 (−0.2197337, 0.4540061)	0.495	1.993	0.736	−0.037	0.660
Mediation effect (XY’): *β*_XY’_ = 0.318, *P*_XY’_ = 0.001224558								

FOLH1, folate hydrolase 1; PHN, postherpetic neuralgia; NAAG, *N*-acetyl-l-aspartyl-l-glutamate; IVW, inverse variance weighting method.

The UVMS analysis also revealed that NAAG was considerably linked with a lower risk of postherpetic neuralgia (*P*_IVW_ < 0.05, *β*_ZY_ = −0.188) in the second step. Moreover, studies have no evidence of horizontal pleiotropy or heterogeneity (*P*_intercept_ = 3.80E-05, *P*_Q_IVW_ > 0.05; see Table 3).

We computed the mediation effect by aggregating the two UVMS step beta values. The results revealed a notable mediation effect (*β*_XY’_ = 0.318, *P*_XY’_ = 0.001224558), so indicating that NAAG most certainly serves as a full mediator between FOLH1 and postherpetic neuralgia.

## Discussion

### Summary of findings

To our knowledge, this is the first study using Mendelian randomization (MR) to extensively assess how metabolites causally affect the risk of postherpetic neuralgia and to investigate their possible roles as mediators in the causal pathway and to fairly evaluate the causal impact of metabolites on PHN risk. Our results show that, showing a clear correlation with a lower risk of PHN, genetically predicted *N*-acetyl-aspartyl-glutamate (NAAG) was the only metabolite to pass multiple corrections. Furthermore, suggestively linked to a higher risk of PHN were gamma-glutamylthreonine, 3-hydroxyphenylacetoylglutamine, Caprate (10:0), X-12013, and X-17328. Furthermore, a mediation MR study verified the NAAG mediating effect between PHN and protein FOLH1. Previous cohort studies have also shown a possible favorable correlation between several metabolites and PHN (12, 41).

### *N*-acetyl-aspartyl-glutamate (NAAG)

Through mGluR3 receptor activation, NAAG seems to alter pain processes. Studies of inhibitors of GCPII, which raise endogenous NAAG levels, show analgesic effects (42, 43). Particularly by means of GCPII inhibitors, Kozikowski et al. (44) and Yamamoto et al. (45) have underlined even more the function of NAAG in neuropathic pain, particularly through the use of GCPII inhibitors. Furthermore shown to produce a prolonged, indirect pain-relieving effect is GCPII inhibition, but the precise mechanisms are yet unknown (43, 46).

Using FOLH1 as the exposure, NAAG as the mediator, and PHN as the outcome, we conducted a mediation MR study to investigate the potential of CGPII inhibitors as targeted analgesics for PHN. Our findings showed that NAAG moderates the interaction between FOLH1 (CGPII) and PHN, implying that higher FOLH1 expression might raise PHN risk. This result implies that possible candidates for focused analgesic treatments for PHN could be CGPII inhibitors.

### Gamma-glutamylthreonine

A byproduct of protein breakdown, gamma-glutamylthreonine acts as a transient intermediary subjecting more proteolysis (47). There is no research in the fields of pain and neuroscience either now or any literature connecting gamma-glutamylthreonine to PHN. This metabolite’s purpose and mechanism of gamma-glutamylthreonine are still mostly unknown and call for more research.

### 3-hydroxyphenylacetylglutamine

Derived from glutamine, 3-hydroxyphenylacetylglutamine results from linking a 3-hydroxyphenyl acetyl group to the glutamine molecule. Individual pain sensitivity and the levels of glutamate and its precursor glutamine showed a positive correlation according to a cross-sectional study in pain-related brain areas (48). Furthermore shown by magnetic resonance spectroscopy is a positive correlation between Central Sensitization Inventory scores and glutamate + glutamine (Glx) concentrations in the thalamus and anterior cingulate cortex (49) and Central Sensitization Inventory scores. These results suggest that 3-hydroxyphenylacetoylglutamine may be involved in pain perception and central sensitization; raised levels of this molecule could be related with PHN. More study is needed to verify its participation in PHN requires detailed investigation in experimental scrutiny.

### Caprate (10:0)

Our Mendelian randomization study suggests a possible causative role for this medium-chain fatty acid in the pathophysiology of neuralgia by pointing up a suggestive correlation between Caprate (10:0) and postherpetic neuralgia.

As a GPR84 agonist, caprate (10:0) has been shown to cause membrane ruffling and motility in microglia without aggravating pro-inflammatory cytokine expression. This suggests that distinct from classical pro-inflammatory responses, microglial activation states could be linked to the aggravation of neuralgia symptoms (50). The absence of classical inflammation implies that microglial activities at the sites of neuronal damage may mediate the pain and hypersensitivity linked with postherpetic neuralgia by means of synaptic remodeling or change of neuronal excitability, which are influenced by the presence and activity of microglia at the sites of neuronal injury.

Conversely, a research indicates that Caprate (10:0) seems to be a possible therapeutic agent and additional alternative able to reduce trigeminal nerve damage sensations in the absence of inflammation/neuropathies (51). From an energy metabolism perspective, Caprate (10:0) is usually regarded as a peripheral metabolic substrate from an energy metabolism standpoint. Via *β*-oxidation, it can pass the blood–brain barrier and be transformed within astrocytes in the brain into acetyl-CoA, then enters the TCA cycle and synthesis of glutamine results from this process, finally producing GABA in neurons (52). We hypothesize that the nerve damage and chronic inflammation brought on by postherpetic neuralgia could indirectly affect cellular energy metabolism and mitochondrial function, so influencing the TCA cycle, reducing GABA synthesis, and affecting inhibitory signal transmission in the nervous system, so influencing the likelihood of neuralgia development. More study is needed to define the precise processes and degree of these effects.

### Strengths

This Mendelian Randomization (MR) study boasts several strengths. To date, this is the most thorough and methodical study into the causal relationship between PHN and 1,091 metabolites. By using different approaches to remove violations of MR assumptions, so addressing reverse causality and confusing factors, our thorough MR analysis reduced possible biases from past studies and produced strong causal estimates. The consistency of our results over sensitivity studies supports even more the dependability of our conclusions. Furthermore, adding to the statistical rigidity of our study is the application of Bonferroni correction for several testing. To verify the validity of the MR estimations, we also performed LD Score Regression (LDSC) and assessed the heritability of the instrumental variables (IVs). Moreover, the possible processes via which metabolites influence PHN was investigated using mediation MR analysis to probe the potential mechanisms through which specific metabolites affect PHN.

### Limitations

One should recognize several constraints. In univariable MR, the assumptions of the two-sample MR design makes linear connections between metabolites and PHN assumption. Future study should include individual-level data for more thorough investigations to handle possible non-linear causal relationships. Furthermore, our dependence on GWAS data predominantly from people with mostly European background reduces the generalizability of the results to different populations. Future research should evaluate these results among several populations to prove more general relevance. Additionally, the possibility of population stratification affecting the results should be considered, and future studies should aim to account for this.

Particularly with regard to PHN severity and specific symptoms, the limited phenotypic detail in the FinnGen GWAS database prevented subgroup studies that might offer more information. Furthermore, the lack of PHN-specific GWAS data limited our capacity to repeat and perform replication and meta-analyses for the metabolites discovered, so verifying the validity of our results.

The rather limited number of SNPs accessible for the exposures of interest at the genome-wide significance level adds still another restriction. We used a relaxed significance threshold, common in such studies, to offset. Still, the *F*-statistic value for every one of the chosen SNPs exceeded 10, so confirming the strength of our instrumental variables (IVs). Fictionally, although MR analysis provides insightful etiological information, our results need validation by means of randomized controlled trials (RCTs) and fundamental research before practical implementation.

A further limitation of this study is the lack of a control group and/or another a group including with other chronic pain disorders. Such diseases future research should incorporate such comparisons should be part of future studies to clarify NAAG’s particular function to further elucidate the specific role of NAAG in PHN and differentiate its effects from those of in other chronic pain conditions.

## Conclusion

In this work, we investigated the causal effect of 1,091 metabolites on the incidence of PHN using a bidirectional MR analysis. Our results show evidence of a causal relationship linking lower risk of PHN to higher degrees of NAAG. On the other hand, suggestively linked with an elevated risk of PHN were gamma-glutamylthreonine, 3-hydroxyphenyl acetylglutamine, Caprate (10:0), X-12013, and X-17328. Moreover, the two-step MR study showed that NAAG performs complete mediation in the link between FOLH1 and PHN. These results provide fresh understanding of the therapeutic processes behind PHN and point out possible directions for next investigation.

## Data Availability

The original contributions presented in the study are included in the article/Supplementary material, further inquiries can be directed to the corresponding authors.
